# Review of Evidence for Using Chest X-Rays for Active Tuberculosis Screening in Long-Term Care in Canada

**DOI:** 10.3389/fpubh.2020.00016

**Published:** 2020-02-07

**Authors:** Mariana Herrera Diaz, Margaret Haworth-Brockman, Yoav Keynan

**Affiliations:** ^1^Grupo de Investigación en Salud Pública, Universidad Pontificia Bolivariana, Medellín, Colombia; ^2^Facultad Nacional de Salud Pública, Universidad de Antioquia, Medellín, Colombia; ^3^National Collaborating Centre for Infectious Diseases, Winnipeg, MB, Canada; ^4^Department of Community Health Sciences, University of Manitoba, Winnipeg, MB, Canada; ^5^Department of Internal Medicine, Medical Microbiology & Infectious Diseases, University of Manitoba, Winnipeg, MB, Canada

**Keywords:** tuberculosis, mandatory testing, nursing homes, cost effectiveness, mass chest X-ray, aged, long-term care, screening

## Abstract

**Background:** People living in long-term care facilities (LTCF) are at high risk to develop active tuberculosis primarily as a result of reactivation of a latent TB infection, or endemic transmission between residents. Current national guidelines in Canada are to use a posterior-anterior and lateral chest X-ray to screen for TB for those over 65 years old, upon admission to a LTCF.

**Objective:** To assess the available evidence for cost benefits of universal chest X-ray screening for new LTCF residents.

**Methodology:** We conducted a search for all articles published until September 2018, in PubMed and WorlCat databases, in English, using a combination of key words: chest X-ray, chest radiography or CXR, long-term care, elderly, screening, and tuberculosis. We also reviewed publicly available guidelines for screening new residents to LTCF from across Canada. We report on a qualitative synthesis of the evidence in the documents retrieved.

**Results:** The final review yielded four cost-effectiveness studies (2 of 4 conducted in countries with low incidence), one systematic review, one recommendation/editorial, and one cohort study. We found that in a tuberculosis low-incidence country the CXR cost per identified case was $672,298 CAD. Enacting a more targeted screening program, perhaps one that tests only those who previously had TB, or other high-risk medical conditions may enhance the cost-effectiveness.

**Recommendations:** We suggest reviewing the screening policy for active TB in people entering LTCF, which is based on a CXR. The results indicate that a targeted search for active TB in people with symptoms or other high-risk medical conditions may be more cost-effective.

## Background

According to the World Health Organization, 9.0–11.1 million people developed tuberculosis (TB) disease in 2017 globally (1). Canada is a low-incidence country, with an incidence rate of 4.9 cases per 100,000 population (1, 2). In 2017, as in previous years, the largest percentage of reported cases in Canada was seen in young adults (aged 25–34 years). However, the highest incidence rate was observed among people aged >75 years, higher than in any other age group at 10.0 per 100,000 population (2). Of active TB diagnoses, 71.8% of cases (*n* = 1,290) were among foreign-born individuals, and 17.4% of cases (*n* = 313) were Canadian-born Indigenous persons (2).

People living in long-term care facilities (LTCF) are considered to have an elevated risk of developing active tuberculosis as a result of (i) reactivation of a latent TB infection (LTBI); (ii) biological (compromised nutrition and immune status, underlying comorbidities, medications-conditions that increase in prevalence with aging) and socioeconomic (poverty, living conditions, and access to health care) factors; and (iii) the close living quarters associated with such facilities (3–5). The *Canadian Tuberculosis Standards, 7th Edition* (2014) includes as LTCF the follow settings: homes for the aged, nursing homes, chronic care facilities, hospices, retirement homes, designated assisted living centers, and any other collective living center (6). Because of the highly communicable potential of the TB bacterium *M. tuberculosis*, transmission between residents and from residents to staff remains a concern in such facilities (3).

According to the *Canadian Tuberculosis Standards 7th Edition*, all new residents entering a LTCF should undergo a history and physical examination by a physician or nurse practitioner to screen for TB within 90 days prior to admission, or within 14 days after admission (6). Active TB screening is conducted using a posterior-anterior and lateral chest X-ray (CXR) upon admission for those over 65 years old, and a baseline two-step TST upon admission for LTBI for identified populations ≤ 65 years old. Verma et al. commented that the implementation of screening LTCF residents varies between provinces (7).

## Objective

The objective of this review was to assess the available evidence for the benefits of the current policies of screening new residents of LTCF using chest X-rays upon admission, and whether such benefits outweigh the risks and/or costs.

## Methods

### Eligibility Criteria

One author (MH) conducted a search and studies were selected according to the following criteria: any article published up to September 2018 in PubMed and WorldCat databases, in English, Spanish or Portuguese. There was no restriction in the countries related to the prevalence of active TB, sex of participants or strategies to compare. We excluded any paper where the X-ray was not included as an evaluated strategy, and studies done in settings other than LTCFs.

### Searching Strategies

We used a combination of keywords: *chest X-ray, chest radiography, CXR, screening, long-term care, tuberculosis*, and *elderly*. The searches were conducted in September 2018 and all articles published before that were reviewed. The searches were replicated independently by a second author (MH-B) to ensure no studies were missed. One additional paper was retrieved in the repeated search.

### Study Selection, Data Collection

Once the articles were identified in the search strategies, we proceeded with the elimination of duplicate items and reading abstracts. Publications selected and related to active TB screening in people who enter or reside in LTCF were read and relevant data were extracted to an Excel worksheet: author(s), article name, year of publication, country and incidence level, study type, sample size and methodology, overview of findings, results and relevance to research question.

With the intention of determining which people are the most important to prioritize for screening, we expanded the search to look for articles that refer to risk populations for active TB among those over 65 years of age. In addition, we conducted an on-line search of websites for the Canadian provinces and territories to determine any available information on current TB screening requirements for LTCF residents. We report on a qualitative synthesis of the collected information.

Ethics approval was not required for this review.

## Results

Table 1 summarizes the results of our on-line search of provincial and territorial requirements or guidelines for chest X-rays to screen for TB in LTCF residents. As the table illustrates, definitions of LTCF vary somewhat, as do priority populations and the timing and methods for TB screening for new residents.

**Table 1:** Current Canadian recommendations for chest X-rays upon admission to long-term care facilities, by province and territory.

**Province/** **Territory**	**Source on TB procedures**	**LTCF definition**	**TB screening on admission**	**Other**
Alberta	Alberta Health and Wellness. Tuberculosis prevention and control guidelines for Alberta Edmonton, AB: Government of Alberta; 2010 https://open.alberta.ca/dataset/ffaa5ef8-dffd-4516-9ffe-0deaa6a945d1/resource/42bcee8b-60cf-442e-a3cb-f2f3cb8c4904/download/tb-prevention-control.pdf	Include supportive living and long-term care accommodations with different levels of care	PA and lateral chest X-ray	Within 6 months of application for admission
British Columbia	BC Center for Disease Control. *Tuberculosis manual. Chapter 4: TB screening and testing*. Vancouver, BC: BC Center for Disease Control; 2015. http://www.bccdc.ca/health-professionals/clinical-resources/communicable-disease-control-manual/tuberculosis	Include long term care, extended care, nursing homes and assisted living each offering different levels of care	<60 X-ray if symptomatic or TST positive >60 X-ray if symptomatic	<60 receive TST to monitor for LTBI
Manitoba	Manitoba Health, Healthy Living, and Seniors. Manitoba tuberculosis protocol. Winnipeg, MB: Manitoba Health, Healthy Living, and Seniors; 2014 https://gov.mb.ca/health/publichealth/cdc/protocol/tb.pdf	Include personal care homes, nursing homes, chronic care facilities, supportive housing and assisted living with different levels of care	Baseline PA and lateral chest X-ray on admission for certain populations	Born before 1955 Aboriginal born or resided in high TB incidence countries
New Brunswick	Government of New Brunswick. Eligibility for publically funded TST and testing policy in Non-Hospital Settings. Fredericton, NB: Government of New Brunswick; 2016. https://www2.gnb.ca/content/dam/gnb/Departments/h-s/pdf/en/CDC/TuberculosisTestingRecommendationsUpdate.pdf	Nursing homes providing 24 h nursing and supervision	No current recommendations on admission	
Newfoundland and Labrador	Government of Newfoundland and Labrador. Guideline for preventing the transmission of *Mycobacterium tuberculosis* across the continuum of care. St. John's, NL: Government of Newfoundland and Labrador; 2015 https://health.gov.nl.ca/health/publichealth/cdc/tuberculosis_management.pdf	Homes for the aged, nursing homes, chronic care facilities, hospices, retirement homes and designated assisted living centers	PA and lateral chest X-ray on admission	
Nova Scotia	Nova Scotia Department of Health and Wellness, Infection Prevention and Control Nova Scotia. Infection prevention and control: guidelines for long-term care facilities. Halifax, NS: Nova Scotia Department of Health and Wellness; 2015 https://ipc.gov.ns.ca/sites/default/files/IPCNS%20Infection%20Prevention%20and%20Control%20LTC%20Final(1).pdf	Nursing homes (24 h care) and residential homes (limited medical care)	>65 with symptoms require chest X-ray <65 high risk populations receive TST and chest X-ray if positive	High Risk Populations Former employees/residents of homeless shelters or correctional facilities Former IV drug users Aboriginal Canadians Positive HIV status People born in high TB incidence countries
Northwest Territories	Northwest Territories Health and Social Services. Tuberculosis. Yellowknife, NT: Northwest Territories Health and Social Services; 2014. https://www.hss.gov.nt.ca/en/services/tuberculosis	Include Long term care and Extended Care offering different levels of care	No current recommendations on admission	
Nunavut	Government of Nunavut. Tuberculosis manual. Iqaluit; NU: Government of Nunavut; 2018 https://gov.nu.ca/sites/default/files/nunavut-tuberculosis-manual-2018.pdf	Include assisted living, residential care and nursing homes	No current recommendations on admission	
Ontario	Ontario Agency for Health Protection and Promotion (Public Health Ontario). Tuberculosis screening on admission to long-term care homes in Ontario. Toronto, ON: Queen's Printer for Ontario; 2019. https://www.publichealthontario.ca/-/media/documents/report-tb-screening-ltch.pdf?la=en	Facilities that provide 24 h nursing and personal care	PA and lateral chest X-ray	Within 14 days of admission unless X-ray done 90 days prior admission
Prince Edward Island	Department of Health and Wellness. (2016). Tuberculin Skin Testing Policy. Chief Public Health Office- Government of Prince Edward Island: 2016. https://www.princeedwardisland.ca/sites/default/files/publications/tuberculin_skin_testing_policy_web.pdf	Nursing homes providing 24 h nursing and supervision	No current recommendations on admission	
Quebec	Ministère de la santé et des services sociaux du Québec. Tuberculose. Québec, QC: Gouvernement du Québec; 2018 http://www.msss.gouv.qc.ca/professionnels/maladies-infectieuses/tuberculose/	Temporary or permanent accommodation facilities that provide 24 h care and supervision; continuing care homes	No current recommendations on admission	
Saskatchewan	Government of Saskatchewan. *Tuberculosis control: a reference guide to the tuberculosis program in Saskatchewan* Saskatoon, SK: Saskatoon Health Region; 2005 https://www.saskatoonhealthregion.ca/locations_services/Services/TB-Prevention/Documents/Resources/Tuberculosis%20Control%20-%20A%20Reference%20Guide%20to%20the%20TB%20Program%20in%20Saskatchewan.pdf	Include nursing homes and special care homes with 24 h nursing supervision	Chest X-ray done on admission	Unless X-ray completed within 90 days of admission
Yukon	Government of the Yukon. (2015). TB Control Manual. Retrieved from: http://www.hss.gov.yk.ca/tbmanual.php	Include long term care and assisted living homes offering different levels of care	No current recommendations on admission	

*TST, tuberculin skin test; PA, posterior-anterior; LTBI, latent tuberculosis infection*.

**Figure 1:**
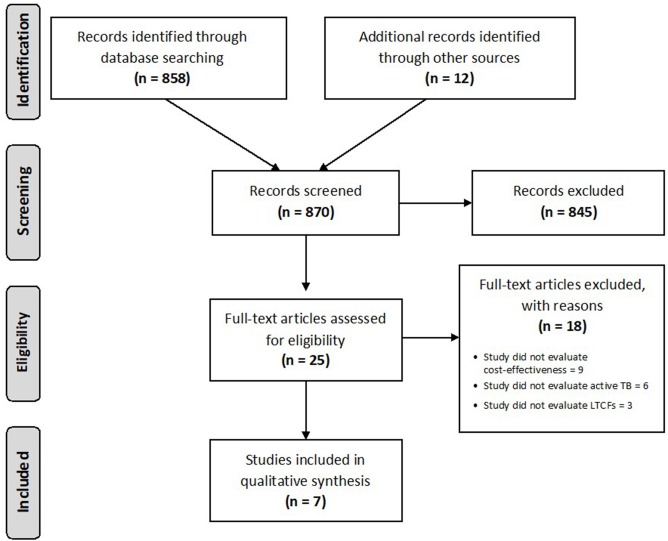
Flow diagram showing the results of searches and articles reviewed. Adapted from PRISMA, Available online at: http://prisma-statement.org/PRISMAStatement/FlowDiagram.aspx.

We found 870 papers in our searches, of which 25 were related to our objective, and only seven studies were relevant (see Figure 1). Of the seven, we found four cost-effectiveness studies (2 of 4 conducted in countries with low incidence) (7–10), one systematic review (11), one retrospective cohort study (12), and one summary of evidence and recommendations (4). The most important results are summarized below and Table 2 shows the detailed information for each study.

**Table 2:** Characteristics and main results of manuscripts that considered chest X-rays for active TB case finding, by study type and year of publication.

**References**	**Country (incidence level)**	**Study type**	**Sample size and methodology**	**Results**
Li et al. (8)	China (Intermediate: (article definition)/(high: WHO definition)	Cost-effectiveness modeling	Derived from Markov model, a decision analytic process was created using an imagined 65-years-old sample and a 20 years time frame. Four different screening strategies were analyzed: (i) no screening, (ii) TB screening (CXR), and (iii) TB screening (Xpert) represent screening for TB in symptomatic elderly by chest X-ray and Xpert® MTB/RIF, respectively, and (iv) LTBI/TB by QuantiFERON®-TB Gold In-Tube and chest X-ray. No screening was the reference. US $50,000 per QALYs gained was used as the threshold for cost-effectiveness.	No screening was the most cost-saving strategy. However, in 1,000 iterations of Monte Carlo simulation, the probabilities of no screening, TB screening (CXR), TB screening (Xpert), and LTBI/TB screening to be cost-effective were 0, 1.3, 20.1, and 78.6%, respectively. Also LTBI/TB screening was the most effective strategy with highest life-years (LYs) and life-years and QALYs.
Kowada (9)	Japan (Low)	Cost-effectiveness modeling	Population studied was comprised of fictitious 84-years-old LTCF residents, some with various comorbidities. Markov models and decision trees were created for seven different screening strategies. Quality-adjusted life-years (QALYs) were used as primary outcome for effectiveness.	The most cost-effective screening practice was QFT (US$ 50,000/QALY), while TST then QFT was best for residents with comorbidities. Diagnosing and treating LTBI is a more efficient procedure than active case-finding, as concerns the mitigation of TB in LTCFs.
Verma et al. (7)	Canada (Low)	Cost-effectiveness modeling	A Markov model was constructed for the three screening strategies to be assessed [no screening, two-step TST (LTBI), and CXR (active TB)] on entry to LTCFs. Alberta LTCF resident TB screenings from 2000 to 2010, and historical charts of TB patients from 1990 to 1996 made up the sample for this analysis. Outcomes for this model were TB cases averted, cases of active TB found with each strategy, and amount saved per case found. One-way sensitivity analysis done for all factors; 95% CIs and reasonable ranges for data-based probabilities and literature-derived values, respectively.	It required 1410 screenings with the LTBI approach to avert one active case, at a cost of $10,9913 CAD; it took 1266 screenings with the CXR approach to avert one active case, at a cost of $67,2298 CAD. Generally speaking, the authors note that both costs required to identify one case are quite high.
Kowada et al. (10)	Japan (Low)	Cost-effectiveness modeling	They constructed a Markov model to evaluate the cost effectiveness one time TB/LTBI screening of those aged over 65 years with QFT (two steps: detection of TB/LTBI by QFT, followed by detection of TB by CXR) vs. one-step detection of TB by CXR, or a no screening strategy, using a hypothetical cohort of 1,000 immunocompetent 65 years-old, vaccinated people. The main outcome measure was quality-adjusted life-years (QALYs) gained, using a lifetime horizon.	The no-screening strategy was the least costly ($US 303.51), while QFT was the most effective (14.6516 QALYs) compared with CXR (14.6477QALYs). QFT may become more cost effective than no screening when the sensitivity of QFT is over 0.89 and the prevalence of disease is higher.
Piccazzo et al. (11)	N/A (N/A)	Systematic Review	A systematic review of the literature concerning the utility of CXR for LTBI screening and uncovering TB cases was carried out, beginning with retrieval of 1,111 articles from PubMed, and concluding with a final review of 67 papers.	To reliably diagnose active TB, CXR must be examined “on the basis of temporal evolution of pulmonary lesions.” Normal CXR is not infrequent for patients presenting with TB symptoms and culture-positive. It is essential that a CXR is carried out after a positive TST/IGRA.
Marciniuk et al. (12)	Canada (Low)	Retrospective Cohort Study	518 patients who were culture-positive for TB were identified between 1988 and 1997. Medical history, symptoms, test results (CXR, cultures), and demographic data were reviewed and explained through descriptive statistics.	Positive cases of TB with normal CXR are not infrequent, and may be on the rise. 25 (4.8%) of the sample classified as having culture-positive TB had regular CXRs; 23 of these 25 had symptoms indicative of TB, while contact tracing was used to diagnose the remaining 2.
Thrupp et al. (4)	United States (low)	Review and position statement	A review of evidence and analysis as background for a position statement and recommendations to prevent and control TB in LTCF. No method described.	Newly admitted patients should undergo TST unless a prior positive result is already on record. A chest radiograph and clinical diagnostic evaluation should be performed for those with a positive TST result. If the initial TST result is negative, the second step of a two-step test should be done. Patients with known prior positive TST results with normal findings on chest radiographs or those with stable old changes on chest radiograph should be re-evaluated periodically for change in symptoms suggestive of TB. Chest radiographs should be repeated only if clinically indicated.

*CXR, Chest X-ray; TB, tuberculosis; TST, tuberculin skin test; IGRA, Interferon-Gamma Release Assays; N/A, Not applicable; QFT, QuantiFERON®-TB Gold In-Tube; QALYs, quality adjusted life-years gained*.

### Cost-Effectiveness of Screening Using CXR to Find Active TB Cases

A 2013 cost-effectiveness study by Verma et al. (7) compared three approaches: no screening, LTBI screening with a baseline two-step TST, and screening for active TB with a chest radiograph. Using a simulation model with real data in Alberta, Canada, the authors found the cost for chest X-rays (CXR) per identified case to be $672,298 CAD. It took 1,266 screenings using CXR to avert one active case, which the authors considered to be “quite high” (7). Their results showed that identification and treatment of latent tuberculosis infection (LTBI) cost $109,913 per case averted but concluded that neither approach is cost-effective. The authors performed a sensitivity analysis and identified that LTBI reactivation has the greatest impact on cost effectiveness of screening, however there is no existing estimate of annual risk for reactivation among the elderly.

Li et al. used a Markov model to assess costs of four different screening strategies for a simulated population of over 65 years old in 2018 (8). Using their pre-determined cost-effectiveness threshold of $50,000 US per QALY gained, screening for TB using CXR was 1.3% effective (with no screening being 0), considerably less cost-effective than screening for LTBI.

Kowada ran a similar modeling experiment in 2016 (also with a threshold for cost-effectiveness of $50,000 per QALY gained) for a hypothetical population of 84 years-old LTCF residents in Japan with previous BCG vaccination. Of seven possible screening strategies, using CXR alone was found to be the least cost-effective means to find new TB cases (9). Also in Japan, Kowada et al. (10) modeled a hypothetical cohort of immunocompetent and 65 years-old people using three different strategies. Their results showed that no TB screening is the most cost-saving strategy, but the QFT is the most cost-effective, assuming high TB/LTBI prevalence (10).

### Assessment of CXR and Other Methods to Screen for TB in LTCF

The single systematic review we retrieved found no consensus on the definition and interpretation of “abnormal chest radiograph consistent with prior TB,” nor on the importance of different sized non-calcified fibrotic lesions (11). Picazzo et al. found that a normal CXR is a frequent occurrence for patients presenting with symptomatic or culture-positive TB (11). For example, in a retrospective cohort study, Marciniuk et al. found that 25/518 (4.8%) of persons screened having culture-positive TB had normal CXRs; 23 of these 25 had symptoms indicative of TB, while contact tracing was used to diagnose the remaining two (12) [and as cited in Piccazzo et al. (11)]. Conversely, in another study that looked at the utility of CXR for diagnosing TB, 159/2,686 CXR exams (6.1%) were determined to have atypical results, yet none of these cases had active TB (13). Picazzo et al. concluded that CXR has good sensitivity but poor specificity for diagnosis of pulmonary TB (11).

When comparing the modeling results for four possible screening strategies, Li et al. found that the most cost-effective strategy was LTBI/TB screening, providing the highest Life Years (LYs) and QALYs gained (8). Results from Kowada's modeling were similar, finding that the most cost-effective TB screening practice was QuantiFERON ($ 50,000 US/QALY), while TST (tuberculin skin test) followed by QFT was best for residents considered to be higher risk of TB reactivation due to comorbidities, such as HIV infection, diabetes mellitus and chronic kidney disease (9).

In their 2004 position paper, Thrupp et al. recommended that newly admitted patients undergo TST unless there is a record of a previous positive result. They recommend CXR and clinical diagnosis for those with a positive TST result, and a second step of a two-step test if initial TST results are negative. The authors recommended repeat TSTs for employees and LTCF residents if new symptoms consistent with TB are observed or if other residents develop TB disease or TST conversions (4).

### Specific Considerations on the Risk of TB for Elderly Women and Men

Several papers point out that co-morbidities that are prevalent in aging populations may further increase the risk of developing active TB disease, including diabetes mellitus and chronic obstructive pulmonary disease (COPD) (4, 14, 15). Canadian data show that ~25% of residents in LTCF have type 2 diabetes and that the number of residents with type 1 diabetes is unknown (16). In 2012–2013, 15% of seniors between the ages of 65 and 69 were living with COPD; among seniors aged 85 years and older, 26% were living with COPD (17). In addition, older patients are more frequently treated with medication that may suppress protective immunity. The most common example of this is corticosteroids in elderly women and men with COPD. Amongst immunosuppressive therapies, anti-TNF therapy particularly increases the risk of active TB (14, 18).

In countries with low TB incidence, migrants from countries with moderate or high TB incidence can be considered a high-risk group (7). In the United States in persons >65 years, the rate ratio of TB incidence comparing foreign-born to US-born persons, was 5.1 (CI95% 5.0–5.2) between 1903 and 2008 (18). A longitudinal study of the incidence of active TB in immigrants arriving between 1975 and 2007 in Victoria State, Australia, found that the risk of active TB was age dependent, with a bimodal peak in incidence among 20–24 years old and 70–74 years old. Region of origin is an important predictor of TB risk; in this study the rates of TB incidence on arrival were similar to the reported incidence rates in the countries of origin (19).

People residing in LTCF may have higher rates of TB than other older adults because they are at risk due to both a predisposition to reactivation of LTBI as well as an increased risk of cross-infection from an index case within the care home environment (4, 20). Careful evaluation of *de novo* infection and reactivation of LTBI may help to stratify risk in patients and target care to those at the highest risk of developing active TB (14), but this requires further exploration.

## Discussion and Conclusions

The findings of this literature search show limited evidence supporting the recommendations for chest X-ray screening. Only one cost effectiveness study was done using Canadian data, and CXR was not found to be cost-effective for active TB screening (7). Three other cost-effectiveness studies determined that CXR was less cost-effective compared with other methods of TB screening (8, 9). Diagnosing and treating LTBI was found to be a more efficient strategy than active case-finding to mitigate TB in LTCF (8).

Recently, Ontario completed a technical report assessing active TB screening at entry to LTCF. The report identified that a very small proportion of LTCF residents in Ontario develop pulmonary TB (incidence rate of 4.6/100,000 per year, 2006–2015), and that LTCF contributes few pulmonary TB cases in Ontario (1.0% on average per year using data from 2006 to 2015) (21). Based on the results and in light of the goal of minimizing case rates and the spread of disease, it appears that a broad screening for every individual above the age of 65 is of low yield and is associated with significant cost. An option of a more targeted screening program, perhaps narrowing the scope of screening for active TB to those with prior TB, known TB exposure, a TST or IGRAs positive, or other high-risk medical conditions can enhance the screening cost-effectiveness (4, 7). There is support in the literature to give consideration to whether residents may be high risk if they have come to a low-incidence country like Canada from countries with high or moderate TB prevalence (14, 18, 19). However, we found no papers that discussed elderly Indigenous women and men who live in LTCF, nor any discussions of the aging populations in Indigenous communities, the care they may need, nor considerations for any heightened risk of re-acquiring or acquiring pulmonary TB. Hochberg and Horsburgh's review of US data suggests that older men, “people of color (including “Native American and Alaska Native),” and those who live in LTCF are at greater risk for active TB, but they do not contextualize their findings with preceding living conditions for those persons, such as life experiences of oppression, racism or poverty (18). Any new explorations of appropriate screening and care for elderly First Nations, Inuit, or Metis women and men in LTCF should be situated in the history of poor health care services and structural and systemic processes that have contributed to exposure to active TB and LTBI (22).

The major limitation of this assessment is the lack of data on elderly TB patients from all provinces and territories, as the prevalence and incidence varies (Manitoba has higher prevalence and incidence of active TB compared to Nova Scotia and Alberta, for example), hence applicability of the analysis is limited. In addition we did not conduct a cost-effectiveness analysis, nor is such analysis available for provinces other than Alberta.

As Canada's populations age, there is on-going need for training for physicians, nurses and other health care workers on TB natural history, disease progression, consideration of TB in the presence of respiratory symptoms in older women and men (4) and culturally appropriate prevention and responses (22). In addition, education for residents and their contacts about TB signs and symptoms is needed (7, 23). In the case of LTCF, these contacts can include staff, patients, family members, volunteers, and visitors (4, 5). A targeted approach for identifying sub-populations of LCTF residents with higher risk for active TB due to epidemiological considerations or the presence of specific co-morbidities, may improve the cost effectiveness of screening.

## Author Contributions

YK and MH-B: substantial contributions to the conception or design of the work. MH and MH-B: acquisition of data. MH, YK, and MH-B: analysis and interpretation of data, drafting the article and revising it critically for important intellectual content, and final approval of the version to be published.

### Conflict of Interest

The authors declare that the research was conducted in the absence of any commercial or financial relationships that could be construed as a potential conflict of interest.
